# Metformin Potentiates the Benefits of Dietary Restraint: A Metabolomic Study

**DOI:** 10.3390/ijms18112263

**Published:** 2017-10-28

**Authors:** Marta Riera-Borrull, Anabel García-Heredia, Salvador Fernández-Arroyo, Anna Hernández-Aguilera, Noemí Cabré, Elisabet Cuyàs, Fedra Luciano-Mateo, Jordi Camps, Javier A. Menendez, Jorge Joven

**Affiliations:** 1 Unitat de Recerca Biomèdica, Hospital Universitari Sant Joan, Institut d’Investigació Sanitària Pere Virgili, Universitat Rovira i Virgili, 43201 Reus, Spain; marta.riera.borrull@gmail.com (M.R.-B.); ghanabel@gmail.com (A.G.-H.); salvador.fernandezarroyo@gmail.com (S.F.-A.); anna.hernandeza@gmail.com (A.H.-A.); noemi.cabre@gmail.com (N.C.); fedra.luciano@gmail.com (F.L.-M.); jcamps@grupsagessa.com (J.C.); 2 Centro de Investigaciones Biológicas (CIB-CSIC), 28040 Madrid, Spain; 3 Molecular Oncology Group, Girona Biomedical Research Insitiute (IDIBGI), 17190 Girona, Spain; ecuyas@idibgi.org (E.C.); jmenendez@idibgi.org (J.A.M.); 4 ProCURE (Program against Cancer Therapeutic Resistance), Metabolism and Cancer Group, Catalan Institute of Oncology, 17190 Girona, Spain; 5 The Campus of International Excellence Southern Catalonia, 43003 Tarragona, Spain

**Keywords:** adipose tissue, caloric restriction, energy state, inflammation, liver steatosis, metabolomics

## Abstract

Prevention of the metabolic consequences of a chronic energy-dense/high-fat diet (HFD) represents a public health priority. Metformin is a strong candidate to be incorporated in alternative therapeutic approaches. We used a targeted metabolomic approach to assess changes related to the multi-faceted metabolic disturbances provoked by HFD. We evaluated the protective effects of metformin and explored how pro-inflammatory and metabolic changes respond when mice rendered obese, glucose-intolerant and hyperlipidemic were switched to diet reversal with or without metformin. Mice treated with metformin and diet-reversal showed a dramatically improved protection against HFD-induced hepatic steatosis, a beneficial effect that was accompanied by a lowering of liver-infiltrating pro-inflammatory macrophages and lower release of pro-inflammatory cytokines. Metformin combined with diet reversal promoted effective weight loss along with better glucose control, lowered levels of circulating cholesterol and triglycerides, and reduced adipose tissue content. Our findings underscored the ability of metformin to target the contribution of branched chain amino acids to adipose tissue metabolism while suppressing mitochondrial-dependent biosynthesis in hepatic tissue. The relationship between adipose tissue and liver might provide clinical potential for combining metformin and dietary modifications to protect against the metabolic damage occurring upon excessive dietary fat intake.

## 1. Introduction

Contemporary high-energy and high-fat diets, coupled with the effect of sedentary lifestyles, are closely related to the convergent epidemics of obesity, insulin resistance, type 2 diabetes (T2D) and non-alcoholic fatty liver disease (NAFLD). These metabolic comorbidities seriously compromise health span and quality of life worldwide [1]. The efficacy of lifestyle modification approaches is low and seriously hampered by relatively unknown cellular metabolic strategies and compensatory pathways induced by reducing fat intake [2,3].

Severe calorie (energy) restriction (CR) is effective, but it seems impractical outside of research settings. Putative mechanisms are attractive, however, because the adoption of a CR-like lifestyle and the typical diets in industrialized countries operate as extreme ends of the same disease–health metabolic spectrum [4,5]. An alternative strategy, scarcely investigated, might combine lifestyle modification with pharmacotherapy, and in this context efficacy is likely for adenosine monophosphate-activated protein kinase (AMPK) activators [6]. Among available drugs, metformin has been suggested to promote protective effects against metabolic diseases with little or no toxicological significance [7,8,9,10]. For example, the combination of metformin and a 70% restriction in calories yielded superior results compared to either treatment alone in diabetic rats [11]. More recently, metformin alone increased energy expenditure and improved liver damage and development of T2D-like state in HFD-fed C57BL6/J mice [12,13]. In mice, taking metformin results in a shift to microbial species with beneficial effects in energy homeostasis [14]. In humans, similar results are likely, and the clinical usefulness of metformin in combination with modest lifestyle modification could be expanded to patients without diabetes with the goal of differentially affecting other medical outcomes [15,16,17,18,19].

How metformin works is not fully understood, but several lines of evidence suggest a critical role in energy homeostasis via a complex picture that includes the effect of nutrients and molecular crosstalk between metabolic organs, especially the liver, gut and adipose tissue [20,21]. Here, we performed an integrated analysis of the metabolic phenotypes occurring upon feeding mice experimental diets with or without metformin treatment using a targeted metabolic approach. We tested the hypothesis that concomitant metformin and simple diet reversal treatment could ameliorate HFD-induced disturbances.

## 2. Results

### 2.1. Effects on the Regulation of Body Weight and Food Intake

**Figure 1:**
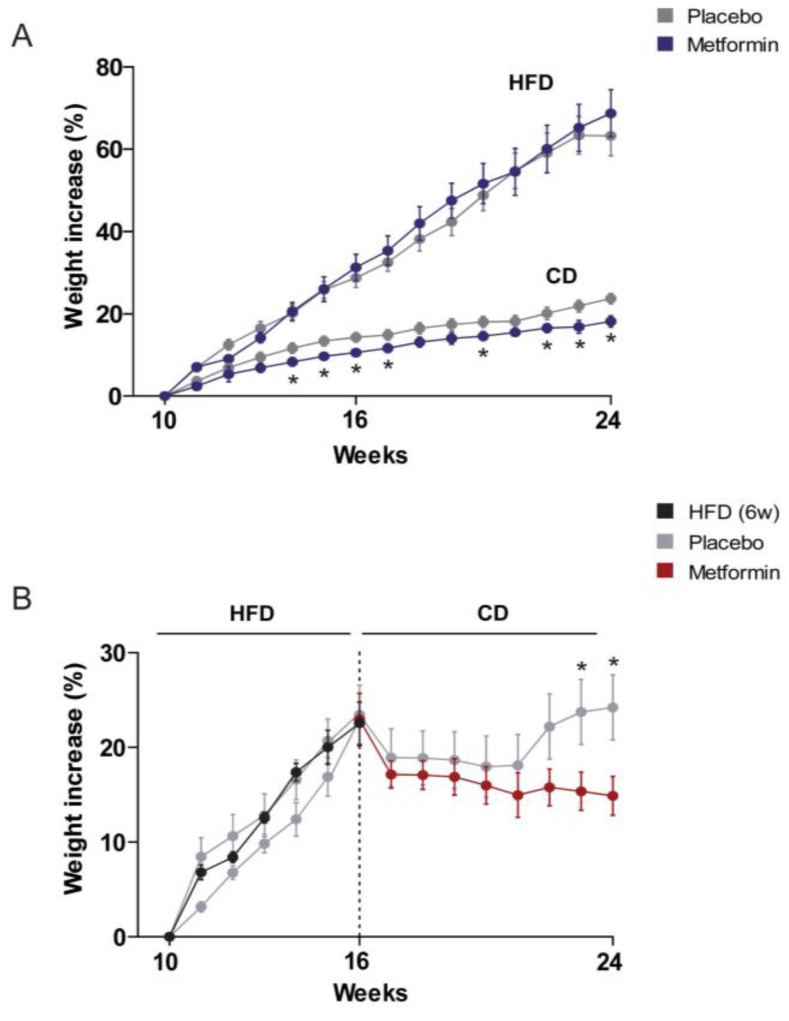
Dietary-induced changes and the effect of metformin on weight control. Overall design in experimental animals. (**A**) Values obtained in mice upon feeding with experimental diets for 14 weeks. CD, chow diet; HFD, high-fat diet; (**B**) values obtained after a 6 week period of HFD feeding and shift to CD. Asterisks denote significant (*p* < 0.05) changes compared to the respective group.

Mice in the HFD group gained weight quicker than chow diet (CD) littermates. Although metformin treatment had no effect on body weight regulation in mice challenged with HFD, it led to a significant reduction in body weight gain in mice on CD from the fourth week of experiment until the end of the follow-up (24 weeks) (Figure 1A). Body weight gain of HFD-fed mice decreased immediately after switching to CD. Remarkably, administration of metformin was significantly more effective than CD alone in reducing body weight gain, particularly after 6–7 weeks of diet reversal when mice fed CD alone began to regain lost body weight (Figure 1B).

**Figure 2:**
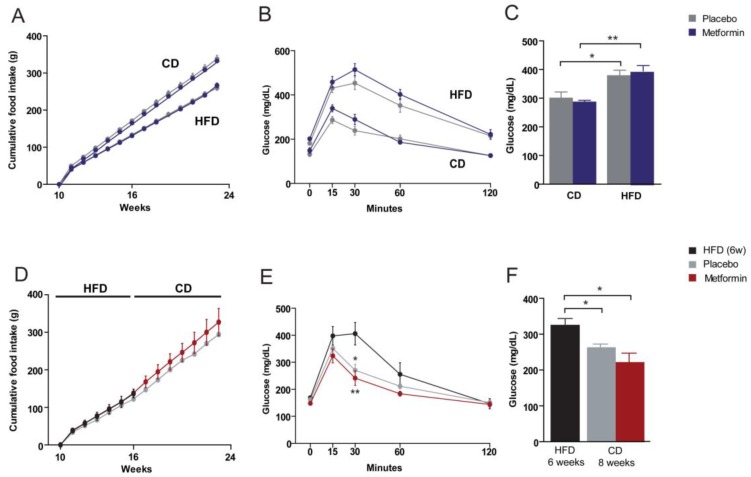
Dietary-induced changes and the effect of metformin on food intake and glucose homeostasis. (**A**,**D**) Cumulative food intake, (**B**,**E**) glucose tolerance tests, and (**C**,**F**) plasma glucose levels segregated by the type of dietary experiment (**A**–**C**) CD versus HFD, and (**D**–**F**) diet reversal and use of metformin as indicated by the legends. Asterisks denote significant (* *p* < 0.05; ** *p* < 0.01) changes.

Despite the gain in weight of HFD mice, the food intake relative to CD-fed-mice was lower. The intake in calories, however, was higher. Data shown in supplementary material indicated differences in energy supply (CD, 3.3 Kcal/g; HFD, 5.7 Kcal/g) per gram of consumed food. The source of these calories was also different. Fat was the main provisor in HFD and carbohydrates in CD. Differences in energy expenditure are unlikely in this model. Metformin treatment had no effect on food consumption between groups (Figure 2A,D).

### 2.2. Effects on Glucose Homeostasis

Fasting serum glucose levels were significantly higher in the HFD group than in the CD group. To assess the impact of chronic HFD exposure on glucose homeostasis in more detail, both groups were subjected to a glucose tolerance test (GTT). Intraperitoneally injected glucose resulted in a more rapid increase of blood glucose in the HFD group than in the CD group, indicating the development of HFD-induced systemic glucose intolerance. The effect of metformin administration on plasma glucose levels and glucose tolerance was negligible in CD and HFD groups (Figure 2B,C). We then re-evaluated glucose homeostasis in HFD-fed mice subjected to diet reversal with or without metformin treatment (Figure 2E). Diet reversal and metformin were similarly effective in reducing the HFD-provoked increase in plasma glucose (Figure 2F). Of note, although peak values of blood glucose levels were similar for diet reversal alone and with metformin in the GTT, animals treated with metformin exhibited faster glucose clearance (Figure 2E).

### 2.3. Effects on Hyperlipidemia

**Figure 3:**
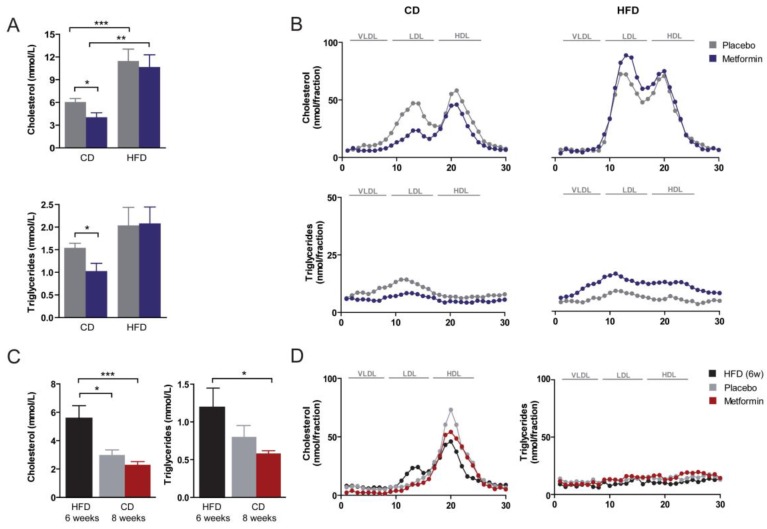
Dietary-induced changes and the effect of metformin on plasma lipids and lipoprotein distribution. (**A**) Plasma lipids and (**B**) lipoprotein distribution as measured with fast-performance liquid chromatography in mice upon feeding with experimental diets for 14 weeks. CD, chow diet; HFD, high-fat diet; (**C**,**D**) same measurements in mice after diet reversal with or without metformin. Asterisks denote significant (* *p* < 0.05; ** *p* < 0.01; *** *p* < 0.001) changes as indicated. LDL: low-density lipoprotein; VLDL: very low-density lipoprotein; HDL: high-density lipoprotein.

Plasma levels of cholesterol and triglycerides were significantly higher in the HFD group than in the CD group (Figure 3A). Metformin treatment resulted in a reduction in the concentration of total serum cholesterol and of dense low-density lipoprotein (LDL) cholesterol in CD-fed animals; an effect not observed in HFD-fed animals (Figure 3A,B). Metformin-induced changes to LDL levels in CD-fed mice were accompanied by a significant decrease in serum triglyceride concentrations and of triglycerides in the LDL fraction (Figure 3A,B). Diet reversal to CD abolished the increase in circulating cholesterol promoted by HFD feeding (Figure 3C). Indeed, lipoprotein distribution in mice subjected to diet reversal with or without metformin largely resembled that commonly observed in wild-type animals (i.e., almost all cholesterol in the high-density lipoprotein (HDL) fraction) (Figure 3D). Administration of metformin promoted a further reduction in the circulating levels of cholesterol in diet-switched mice, and also a statistically significant reduction in serum triglyceride concentration (Figure 3C). 

### 2.4. Effects on White Adipose Tissue

**Figure 4:**
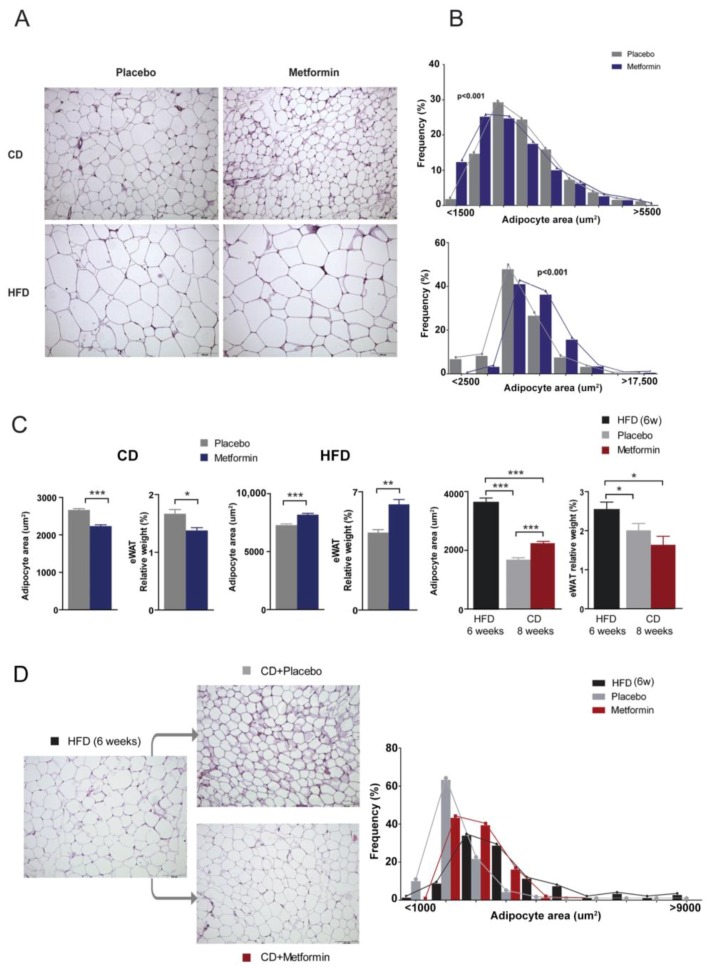
Dietary-induced changes and the effect of metformin on the epididymal WAT phenotype. (**A**) Representative microphotographs (200×) showing the differential effects of diet and metformin in epididymal white adipose tissue (eWAT) and (**B**) frequency distribution of adipocyte size; (**C**) mean values for adipocyte area and relative weight with respect to body weight in experiments; (**C**,**D**) phenotypic changes and frequency distribution of adipocyte size combining shift in diets and metformin. Legends as in Figure 1 and asterisks denote significant (* *p* < 0.05; ** *p* < 0.01; *** *p* < 0.001) changes as indicated.

Given the changes in triglyceride levels between CD and HFD groups, we next questioned how metformin might impact epididymal white adipose tissue (eWAT) content. Morphometric analysis of eWAT depots revealed that average adipocyte size in HFD-fed mice was significantly greater than that found in CD-fed mice (Figure 4A,B). Metformin administration significantly decreased adipocyte size and also the relative weight of the eWAT depot in CD-fed mice. By contrast, metformin treatment further increased adipocyte area and also the relative weight of white adipose tissue (WAT) in HFD-fed animals (Figure 4C). Although metformin treatment augmented the loss of the WAT depot in animals subjected to diet reversal and led to a significant decrease in adipocyte area, the average size of adipocytes remained larger than those from animals treated with diet reversal alone (Figure 4D).

### 2.5. Effects on Hepatic Steatosis

**Figure 5:**
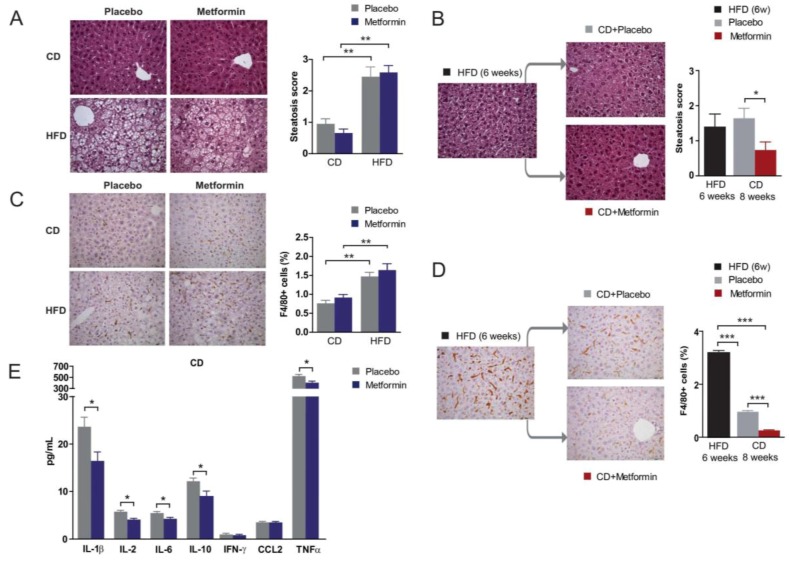
Dietary-induced changes and the effect of metformin on the liver phenotype. Effects of diet and metformin at indicated times during nutritional variations. Representative microphotographs (100×) of liver sections stained with H&E (**A**,**B**) and F4/80 immunochemistry (**C**,**D**) showing the effects of diet and metformin in steatosis score and proportion of macrophages; (**E**) the effect of metformin in the hepatic concentration of selected cytokines in CD-fed mice. Legends as in Figure 1 and asterisks denote significant (* *p* < 0.05; ** p < 0.01; *** *p* < 0.001) changes as indicated.

Chronic HFD exposure is known to cause lipid accumulation in the liver, a process that ultimately leads to NAFLD and eventually to nonalcoholic steatohepatitis. As expected, HFD-fed animals presented with severe fatty liver disease. No significant differences in hepatic steatosis were found in the CD and HFD groups following metformin administration (Figure 5A). Remarkably, whereas diet reversal alone failed to ameliorate HFD-induced hepatic steatosis, the combination of diet reversal and metformin treatment significantly reduced the occurrence of steatosis (Figure 5B).

### 2.6. Effects on Hepatic Inflammation

There were no signs of ballooning or fibrosis and liver enzymes were not differentially affected by diet, but we detected a significantly increased number of macrophages in liver of HFD-fed animals when compared with CD-fed animals, as revealed by immunohistochemistry for F4/80, a pan-marker for murine tissue macrophages (Figure 5C). Diet reversal ameliorated liver inflammation in the HFD group, as demonstrated by the significant decrease in the number of F4/80+ inflammatory cells (Figure 5D). Of note, metformin treatment of diet-reversed animals almost completely eliminated the presence of F4/80+ macrophages (Figure 5D), which also appeared to be enlarged; a morphology that might be suggestive of an activated state following HFD. The ability of metformin to reduce HFD-induced chronic inflammation might involve the reduced production of prototypical pro-inflammatory cytokines including interleukin (IL)-1β and tumor necrosis factor-α (TNFα), as levels of these cytokines were significantly reduced in metformin-treated mice. Changes were similar in both CD and HFD regimens. For clarity, only values obtained in CD-fed mice with or without metformin are shown in Figure 5E.

### 2.7. Effects on the Levels of Energy Metabolites in Hepatic and Adipose Tissue

**Figure 6:**
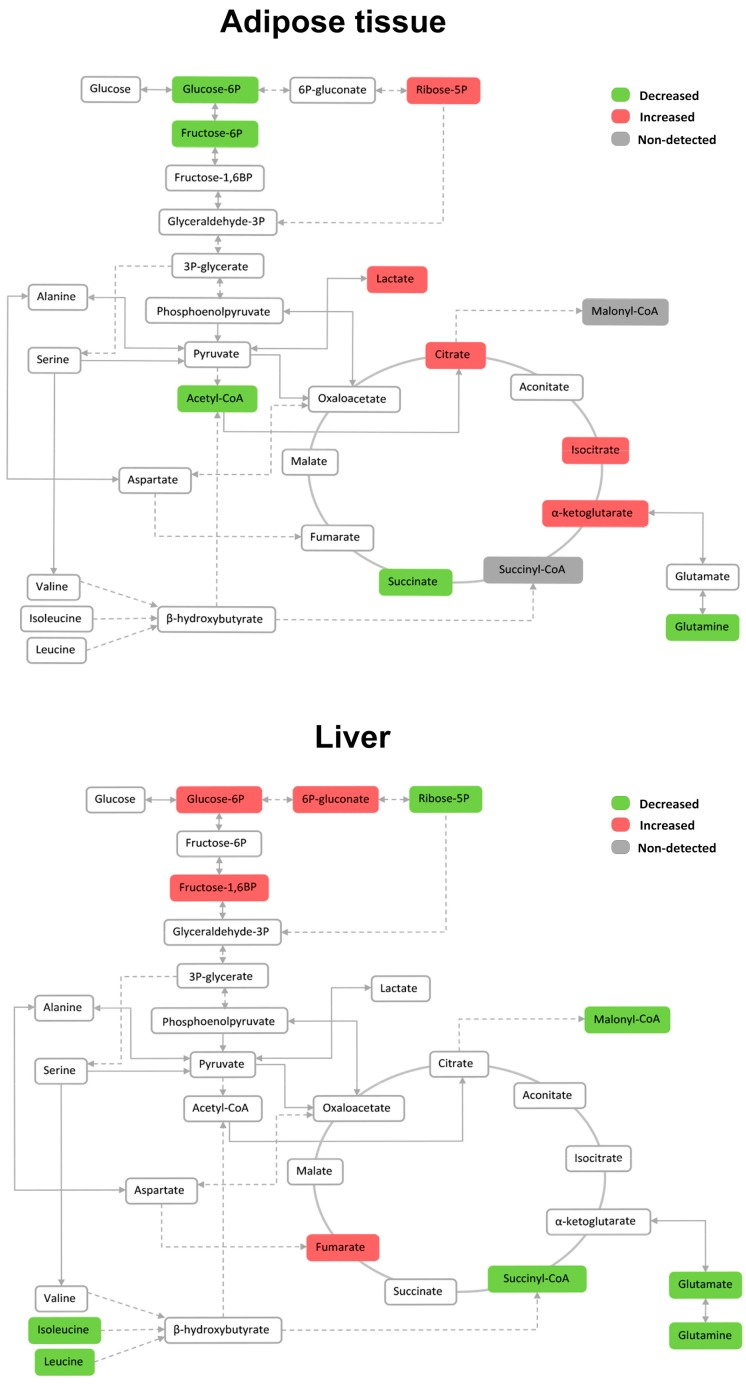
The relative impact of dietary fat on the levels of metabolites associated with energy metabolism in adipose tissue and liver. Metabolites are marked in green, red or grey if they were significantly decreased, increased or non-detected respectively according to the impact of HFD.

The exposure of mice to HFD has an impact on levels of energy metabolites, which is different in WAT and liver. In WAT, HFD led to a decrease in the levels of glycolytic intermediates proximal to glucose-6-phosphate, while increasing the citric acid cycle (CAC) intermediates citrate/isocitrate and α-ketoglutarate. Conversely, HFD promoted the accumulation of glycolytic intermediates proximal to glucose-6-phosphate in liver, that is, those intermediates involved in glucose transport and phosphorylation, while significantly decreasing branched-chain amino acids (BCAAs) (Figure 6).

**Figure 7:**
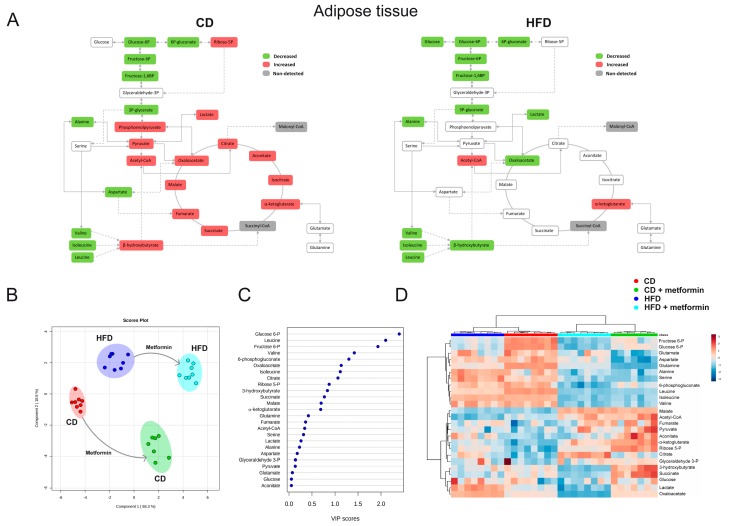
Dietary-induced changes and the effect of metformin on white adipose tissue (WAT) energy metabolites. (**A**) The effect of both diets is segregated to highlight specific changes in metformin response; metabolites are marked in green, red or grey if they were significantly decreased, increased or non-detected respectively; (**B**) partial least square discriminant analysis indicates visually the role of the measured metabolites in discriminating among the different experimental groups; (**C**) random forests were used to rank the importance of metabolites without supervision, in order to explain the differential effect of metformin; (**D**) standardized metabolite concentrations represented as a heatmap. Mice populations with different diets and metformin treatment are reported as a color code in the upper part of the graph, while metabolite names have been assigned to rows.

We then evaluated the tissue-specific (WAT versus liver) nature of the impact of metformin for energy metabolites. Metformin treatment led to a significant decrease in the levels of the BCAAs valine, leucine and isoleucine in WAT of mice fed CD, and additionally increased glycolytic intermediates distal to glucose-6-phosphate (phosphoenolpyruvate, pyruvate and lactate) and most of the CAC intermediates (Figure 7A). Although the decrease in the glycolytic intermediates proximal to glucose-6-phosphate remained unaltered by metformin treatment, neither glycolytic intermediates distal to glucose-6-phosphate nor CAC intermediates were significantly altered by metformin in WAT of mice on HFD. Metabolite-based clustering in WAT, obtained by the partial least squares-discriminant analysis (PLS-DA) model, revealed a clear and significant separation between CD and HFD regimens in the absence or presence of metformin treatment in two-dimensional score plots (Figure 7B). When the criteria of variable importance in the projection (VIP scores ≥1) in the PLS-DA model were used to maximize the difference of metabolic profiles between the different diet groups, the subset of metabolites majorly impacted were early glycolytic intermediates and BCAAs (Figure 7C). Heatmap visualization, commonly used for unsupervised clustering, revealed a similar segregation of metformin-impacted glycolytic and amino acids metabolites in WAT from CD and HFD groups (Figure 7D). 

**Figure 8:**
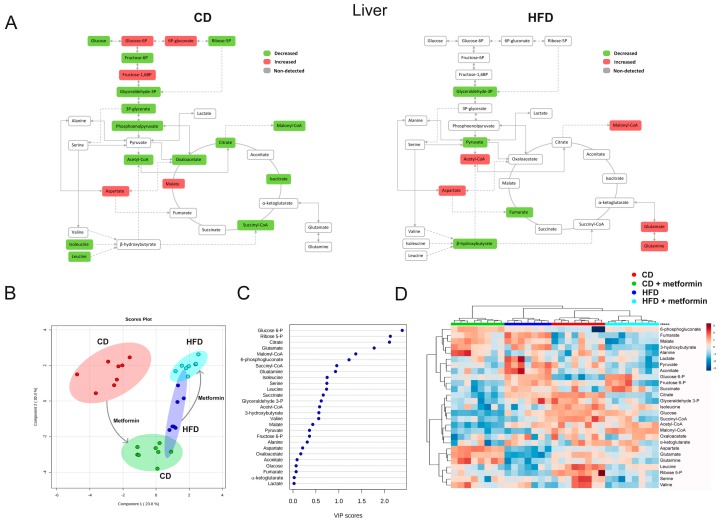
Dietary-induced changes and the effect of metformin on hepatic energy metabolites. Descriptive details are conserved as in Figure 7.

A completely different picture emerged when assessing metformin-driven metabolomic shifts in liver from CD and HFD groups. The significant reduction of phosphoenolpyruvate and acetyl-CoA, accompanied by an increase in the levels of glycolytic intermediates proximal to glucose-6-phosphate, including glucose-6-phosphate itself, 6-phosphogluconate and fructose 1,6-bisphosphate, suggested a reduced entry of glucose carbon into mitochondrial biosynthetic metabolism in mice fed CD together with metformin treatment (Figure 8A). Mitochondria from livers of mice fed HFD apparently exhibited an increased dependency in reductive glutamine metabolism capable of replenishing the high levels of lipogenic acetyl-CoA/malonyl-CoA as an adaptive response to metformin (Figure 8A). Metabolite-based clustering in liver obtained by the PLS-DA model confirmed a clear and significant separation of animals fed a CD alone and with metformin (Figure 8B). Conversely, a small overlap occurred in animals fed HFD alone and with metformin (Figure 8B). When the distinct metabolites between hepatic groups were selected with the criteria of VIP ≥1 in the PLS-DA model, the subset of metabolites majorly impacted were glycolytic intermediates and those metabolites related to the biosynthetic fates of glutamine metabolism (Figure 8C). Unlike the scenario observed with the similar impact of metformin for WAT metabolites in CD- and HFD-fed mice, the segregation of the selected metabolites obtained by using heatmap visualization confirmed a completely distinct segregation of metformin-impacted metabolites in liver of CD- and HFD-fed mice (Figure 8D).

## 3. Discussion

Metformin treatment might play a major role in preventing the metabolic disturbances caused by exposure to HFD in both adipose and liver tissues, and might enlarge the benefits of simple dietary restrain. Feeding mice with HFD promoted the appearance of classical metabolic derangements occurring after excessive intake of dietary lipids. When HFD-fed animals were subjected to diet reversal alone, and also to metformin for a short duration, it became apparent that switching to regular feeding alone could not fully reverse the metabolic abnormalities provoked by the initial exposure to HFD. However, the addition of metformin treatment to diet reversal ameliorated the metabolic status associated with the previous HFD pattern. Animals started to regain body weight six weeks after switching to normal diet, but the trend towards lower weight gain remained unaltered in the presence of metformin, likely indicating that the effect of metformin in weight loss maintenance could prevent the long-term consequences of HFD-associated overweight. The combination between HFD and metformin did not improve glucose tolerance. Our results suggest mechanisms related to insulin signaling. Future research should take into consideration the combined effects of both insulin and metformin [22,23]. Metformin also decreased the circulating levels of cholesterol and triglycerides, revealing its capacity to protect from the long-term effects of HFD-imposed periods of metabolic hyperlipidemia [24]. 

Exposure to HFD causes chronic inflammation in the liver, a critical factor for the development of obesity-related glucose intolerance, insulin resistance and NAFLD progression [25]. This key mechanistic component of the lifelong risk for developing metabolic complications in response to energy overload is affected by metformin. Mice treated with metformin and diet-reversal exhibited an improved protection against HFD-induced hepatic steatosis, which was accompanied by a significant lowering of liver-infiltrating pro-inflammatory macrophages as well as lower levels of major pro-inflammatory cytokines. The ability of metformin to modulate the polarization or migration of these cells has been recently demonstrated in tumor-associated macrophages [26]. Future studies should examine whether metformin contributes to the reduction of hepatic and adipose tissue inflammation by preventing either the recruitment or pro-inflammatory activation of macrophages (or both) [27].

Diet reversal failed to restore HFD-induced hepatic damage unless combined with metformin. Metformin decreased the flow of glucose- and glutamine-derived CAC intermediates and appears to overcome the hepatic ability to store and metabolize carbohydrates, thereby better preventing HFD-induced hepatic damage when switching to normal diet occurs. Mechanisms remain to be fully understood, but are possibly related to known effects on mitochondrial-dependent biosynthetic activity and de novo lipogenesis [28,29]. Hence, elevated mitochondrial CAC function and increased flow through anabolic pathways play crucial roles in the pathobiology of fatty liver and insulin resistance induced by an obesogenic diet [30,31]. This study does not provide clear evidence on how metformin and diet reversal may share mechanisms, but suggests that metformin might alleviate inflammation and that mitochondrial adaptations might be explored as mechanisms of HFD-induced hepatic complications.

It appears that adipocyte hypertrophy, a hallmark of WAT enlargement in obesity, is corrected upon diet reversal. The frequency distribution of adipocyte areas across the WAT depot suggests an apparently limited capacity of metformin to remodel WAT tissue architecture, likely indicating a specific protection from hepatic insulin resistance rather than a protection from systemic insulin resistance. This effect of metformin is diet-specific and likely represents a defense mechanism to protect the liver [32]. Recent data suggest that impaired adipogenesis is associated with insulin resistance [23]. The combined administration of metformin and insulin might provide mechanistic clues to support the synergic effect of metformin and dietary restraint. Future research should include the effect of metformin converging in mechanisms involving BCAAs. We now know [33,34] that differentiated adipocytes exhibit an increased catabolic flux of BCAAs, such as leucine and isoleucine, that account for a significant amount of lipogenic acetyl-CoA pools. In this regard, the capacity of metformin to target the contribution of BCAAs to WAT metabolism is noteworthy. By altering the functional integrity of this tissue, the up-regulation of the BCAA degradation pathway might contribute to the beneficial effects elicited by metformin beyond those of diet reversal alone. The increased degradation of BCAAs may help in maintaining energy homeostasis and glucose homeostasis providing substrates for the tricarboxylic acid cycle. The increased use of BCAA may also indicate an increased protein synthesis.

Although there is still controversy about the mechanisms of action of metformin [35], their health-promoting effects are largely viewed as the consequence of its ability to simultaneously target the core nutrient-sensing networks insulin/IGF-1, at the non-cell autonomous level, and AMPK/mTOR, at the cell-autonomous level [7,36,37,38]. Alternatively, we do not discard primary downstream consequences on a single master mechanism that has not yet been identified. We have found robust patterns indicating the potential of metformin to serve as a model compound for preventing HFD-driven progression of metabolic complications. The clinical combination of metformin-based pharmacotherapy with dietary modification might herald the development of much-needed therapeutic and preventive strategies against metabolic consequences of excessive dietary fat intake. 

## 4. Materials and Methods

### 4.1. Animals, Animal Care and Dietary Details

Low-density lipoprotein receptor-deficient mice (*Ldlr*^−/−^) were used. We have previously shown that this is a robust model to assess the effect of nutrition in a context of subclinical chronic inflammation, which reproducibly measure objective and quantifiable metabolic characteristics resembling metabolic syndrome and includes hyperlipidemia without the confounding effects of diabetes [39,40]. Male Ldlr^−/−^ mice in a C57BL/6 genetic background were obtained by breeding animals purchased from Jackson Laboratory (Bar Harbor, ME, USA) and maintained under controlled temperature (22 °C), humidity (50%) and lighting (12 h–­12 h light-dark cycle). To prevent sex-dependent variability, female animals were not included [39]. All procedures were performed by dedicated staff in accordance with current regulations and supervision by the Ethics Committee on Animal Experimentation of the Universitat Rovira i Virgili (protocol GC-URV-0235-03.18.2014) following European guidelines (Directive 2010/63/EU). Mice had *ad libitum* access to water and control breeder chow diet (CD, calories (3.3 Kcal/g) were from protein (19%), fat (roughly 9%) and carbohydrates (72%)) prepared by Scientific Animal Food & Engineering, Augy, France, until 10 weeks of age and throughout the experiments. The effects of CD were compared to those obtained with a high-fat diet (HFD) prepared by Ssniff Spezialdiäten (Soest, Germany), in which calories (5.7 Kcal/g) were from protein (19%), fat (roughly 60%) and carbohydrates (21%). Additional information on supplied nutrients may be found in Supplementary Table S1.

### 4.2. Experimental Design and Metformin Provision

The animals were allocated to experimental groups using computer-generated randomization schedules, and the investigators responsible for the assessment of outcomes had no knowledge of the experimental group to which the animals belonged. No animals were excluded from the analysis. We first explored the metformin response in mice fed with either the original CD or HFD for 14 weeks. Mice were allocated into two dietary groups (*n* = 16, each) and further divided in mice receiving metformin (Sigma, Madrid, Spain) or placebo (Monteloeder, Elche, Spain) daily (*n* = 8, each). Mice were sacrificed at 24 weeks using isoflurane inhalation. We proceeded similarly in parallel experiments to examine the metformin response in mice with adverse inflammatory and metabolic status established through feeding animals with HFD for 6 weeks. For comparative analysis, some animals (*n* = 6) were sacrificed at this time and other littermates were then fed with HFD or CD for other 8 weeks. Those mice fed with CD were divided to receive metformin or placebo (*n* = 8, each) to assess whether metformin potentiates the effects of switching to CD. Mice were sacrificed at 24 weeks after a similar fasting time (4 h with a maximum difference of 15 min).

We used metformin dissolved in drinking water (5 mg·mL^−1^) to provide 250 mg·kg^−1^·day^−1^. As previously described, mean plasma concentrations in mice were similar to that obtained in humans with a dose of 1.25 g·day^−1^ [36]. Metformin did not accumulate in plasma after continued administration. The choice of this route of administration was based in previously published data [32,41]. In our hands, the action of metformin might be dose- and time-dependent, because unbalanced plasma concentrations of metformin were observed when metformin was given by either bolus gavage or intra-peritoneal injection. We did not achieve sufficient reproducibility when adding metformin to the diets during the manufacturing process and the drug was mostly but irregularly consumed during the night. 

### 4.3. Sample Collection, Analytical Methods and Histological Analysis

Glucose tolerance tests (GTT) were performed in all animals one week before their sacrifice by injecting intraperitoneally glucose in saline solution (2 mg·g^−1^ of body weight) after 4 h of fasting. Blood was drawn from the tail to measure glucose levels (Roche Diagnostics, Basel, Switzerland), 0, 15, 30, 60 and 120 min after the injection. Blood samples were also collected from anesthetized animals into EDTA-treated blood collection tubes during sacrifice and immediately centrifuged and stored at −80 °C until analysis. There were no detectable iatrogenic experimental variables. Plasma concentrations of glucose, cholesterol, triglycerides and liver enzymes were measured by standard assays in an automated analyzer Synchron LXi 725 (Beckman Coulter, IZASA, Barcelona, Spain). Plasma lipoproteins were separated by fast-performance liquid chromatography (FPLC) using a Bio-Rad Bio Logic Duo Flow 10 system (Bio-Rad, Alcobendas, Spain). Serum pooled samples from each group (200 µL) were injected into a Superose 6/300 GL column (GE Healthcare Europe GmbH, Munich, Germany), and 500 µL fractions were collected. Cholesterol and triglycerides concentrations were analyzed in each fraction as reported [39]. Organs were perfused in phosphate buffered saline. Fractions of the liver were homogenized using a Precellys 24 homogenizer (Bertin Technologies, Toulouse, France) in lysis buffer containing protease inhibitors at a concentration of 250 µg/mL. The levels of interleukin (IL)-1β, IL-2, IL-6, IL-10, interferon-γ, chemokine (C-C motif) ligand 2 (CCL2), and tumor necrosis factor-α (TNFα) were measured in the homogenates following the manufacturer’s instructions using Bio-plexProTM magnetic bead-based assays (Bio-Rad, Madrid, Spain) on the Luminex platform (Bio-Rad). Other removed tissue samples such as liver or epididimal white adipose tissue were removed, weighed and stored at 80 °C until needed. For histological examination, tissue samples were fixed for 24 h in 10% neutral-buffered formalin, paraffin-embedded and stained with hematoxylin and eosin (H&E). The hepatic macrophages (anti F4/80, Serotec, Oxford, UK) were examined by immunohistochemistry. The extent of steatosis in the liver, the proportion of F4/80 stained cells and the adipocyte size of epididymal white adipose tissue (eWAT) were estimated by image analysis software (AnalySIS, Soft Imaging System, Munster, Germany) essentially as described [42,43].

### 4.4. Targeted Metabolomics

The list of metabolites of major pathways related to energy metabolism was designed with the rationale that cells must allocate nutrients toward boosting glycolysis, and to generate adenosine triphosphate (ATP) and intermediates for macromolecule biosynthesis. Metabolites were quantitatively measured as described [40]. Briefly, metabolites in liver (50 mg) and epididymal white adipose tissue (eWAT; 100 mg) were extracted in 1 mL of methanol/water (8:2) using a Precellys 24 system (Izasa, Barcelona, Spain). After centrifugation at 14,000 rpm 10 min at 4 °C, supernatants were collected and the homogenization step repeated. Nonpolar compounds were further extracted in chloroform/methanol (2:1). All supernatants were centrifuged, filtered using 0.22 μm filters, and freeze-dried overnight. Samples were dried under N_2_, derivatized using methoxyamine dissolved in pyridine (40 mg/mL) and *N*-methyl-*N*-(trimethylsilyl)-trifluoroacetamide and injected into a 7890A gas chromatograph coupled with an electron impact source to a 7200 quadrupole time-of-flight mass spectrometer (MS) equipped with a 7693 auto-sampler module and a J&W Scientific HP-5MS column (30 m × 0.25 mm, 0.25 μm) (Agilent Technologies, Santa Clara, CA, USA). We measured 31 metabolites in related pathways, including the pentose-phosphate, glycolysis and gluconeogenesis, citric acid cycle (CAC) and metabolism of amino acids (see Figure 6, Figure 7 and Figure 8). Values in eWAT homogenates were lower than in liver. In particular, malonyl-CoA and succinyl-CoA in eWAT were not considered because, although detected, they were under the limit of quantification using these conditions. 

### 4.5. Statistical Analysis

All results are shown as the mean ± SD unless otherwise stated. Differences between groups were assessed with the Mann–Withney *U* test (nonparametric) and considered statistically significant when *p* < 0.05. Some comparisons required one-way ANOVA. All statistical analyses were carried out using the GraphPad Prism 6.0 Software, Inc. (La Jolla, CA, USA). For the metabolomic analysis, the obtained raw data were processed and compounds were detected and quantified using the Qualitative and Quantitative Analysis B.06.00 software (Agilent Technologies). Results were compared by one-way ANOVA with Dunnett’s multiple pair-wise comparison tests using a significance threshold of 0.05. MetaboAnalyst 3.0, available on the web (Available online: http://www.metaboanalyst.ca/) was used to generate meaningful scores/loading plots [44].

## 5. Conclusions

A metformin-induced response may assist in the prevention of the metabolic consequences of obesogenic diets and potentiate the benefits of dietary restraint. Metformin effects are most notably observed in the development and progression of NAFLD. In adipose tissue, metformin targets the contribution of BCAAs, most likely by up-regulating their degradation. Future research should include elucidating the mechanisms by which metformin mediates global epigenetic programming in metabolic tissues, ascertaining the effect of activating AMPK in skeletal muscle and to assessing the likely additive effects with fasting-mimicking diets.

## Supplementary Materials

Supplementary materials can be found at www.mdpi.com/1422-0067/18/11/2263/s1.
