# Supplementary material for: Metformin Potentiates the Benefits of Dietary Restraint: A Metabolomic Study
Source: Int J Mol Sci. 2017 Oct 28;18(11):2263. doi: 10.3390/ijms18112263 (PMC5713233; doi:10.3390/ijms18112263)
Supplement: Supplementary file 1 [file ijms-18-02263-s001.pdf]

Supplementary material corresponding to the manuscript:

# Metformin potentiates the benefits of dietary restraint: a metabolomic study

Marta Riera-Borrull [1,2], Anabel García-Heredia [1], Salvador Fernández-Arroyo [1,3], Anna Hernández-Aguilera [1], Noemí Cabré [1], Elisabet Cuyàs [3,4], Fedra Luciano-Mateo [1], Jordi Camps [1], Javier A. Mendendez [3,4] and Jorge Joven [1,5*].

## Supplementary material and methods S1: Food intake measurement

Food intake measurement was performed as previously reported [1]. Mice were group housed and given a known, pre-weighed amount of food in their home cage hopper. Once a week, the remaining food is weighed. Then, new amount of food is administered. Calculations were performed as follow: Food intake = Food provided – Food ingested – food wasted. Food intake measurements were divided by the number of animals per home cage, so assuming a mean-intake per animals, and represented in g, although it can be easily converted to kcal, by using data on Table S1.

*[1] Ellacott KLJ et al., Cell Metab, 2010.*

## Table S1: Nutritional composition of experimental diets..

| | CD (Chow diet) | HFD (High-fat diet) |
|---|---|---|
| **Crude Nutrients** | | |
| Crude protein (%) | 16.1 | 24.10 |
| Crude fat (%) | 3.1 | 34.60 |
| Crude fibre (%) | 3.9 | 7.2 |
| Carbohydrate (available) (%) | 71.8 | 24.80 |
| Crude ash (%) | 4.2 | 8.5 |
| Nitrogen free extracts (%) | 43.5 | 23.30 |
| **Energy** | | |
| Energy density (Kcal/g (Kj/g)) | 3.3 (13.97) | 5.70 (24) |
| Calories from protein (%) | 19.3 | 19 |
| Calories from fat (%) | 8.4 | 60 |
| Calories from carbohydrate (%) | 72.4 | 21 |
| **Minerals** | | |
| Calcium (%) | 1.38 | 0.98 |
| Phosphorus (%) | 0.99 | 0.65 |
| Sodium (%) | 0.47 | 0.20 |
| Magnesium (%) | 0.34 | 0.17 |
| Potassium (%) | 1.45 | 0.98 |
| Chloride (%) | 0.69 | - |
| **Fatty acids** | | |
| Caprid acid (C10:0) (%) | ND | 0.04 |
| Lauric acid (C12:0) (%) | ND | 0.07 |
| Myristic acid (C14:0) (%) | ND | 0.44 |
| Palmitic acid (C16:0) (%) | 0.87 | 7.72 |
| Palmitoleic acid (C16:1) (%) | 0.03 | 0.94 |
| Margaric acid (C17:0) (%) | ND | - |
| Stearic acid (C18:0) (%) | 0.08 | 4.34 |
| Oleic acid (C18:1) (%) | 0.53 | 13.57 |
| Linoleic acid (C18:2) (%) | 1.34 | 4.75 |
| Linolenic acid (C18:3) (%) | 0.15 | 0.51 |
| Arachidic acid (C20:0) (%) | ND | 0.02 |
| Paullinic acid (C20:1) (%) | ND | 0.01 |
| Arachidonic acid (C20:4) (%) | ND | 0.53 |
| Cholesterol (mg/kg) | ND | 265 |
| **Amino acids** | | |
| Lysine (%) | 3.9 | 1.98 |
| Methionine (%) | 1.49 | 0.83 |
| Cystine (%) | 1.08 | 0.46 |
| Met+Cys (%) | ND | 1.28 |
| Threonine (%) | ND | 1.07 |
| Tryptophan (%) | 0.88 | 0.31 |
| Arginine (%) | 4.75 | 0.88 |
| Histidine (%) | ND | 0.76 |
| Valine (%) | ND | 1.64 |
| Isoleucine (%) | ND | 1.25 |
| Leucine (%) | ND | 2.36 |
| Phenylalanine (%) | ND | 1.29 |
| Phe+Tyr (%) | ND | 2.57 |
| Glycine (%) | 0.41 | 0.50 |
| Glutamic acid (%) | ND | 5.41 |
| Aspartic acid (%) | ND | 1.79 |
| Proline (%) | ND | 2.76 |
| Alanine (%) | ND | 0.79 |
| Serine (%) | ND | 1.43 |
| **Vitamins** | | |
| Vitamin A (IU/kg) | 7500 | 15000 |
| Vitamin $D_3$ (IU/kg) | 1000 | 1500 |
| Vitamin E (IU/kg) | 120 | 225 |
| Vitamin K (as menadione) (mg/Kg) | 2.5 | 20 |
| Vitamin C (mg/Kg) | ND | 30 |
| Thiamin ($B_1$) (mg/Kg) | 7 | 16 |
| Riboflavin ($B_2$) (mg/Kg) | 6.5 | 16 |
| Pyridoxime ($B_6$) (mg/Kg) | 2.6 | 18 |
| Cyanocobalamin ($B_{12}$) (µg/Kg) | 20 | 30 |
| Nicotinic acid (mg/Kg) | 75 | 45 |
| Pantothenic acid (mg/Kg) | 17 | 55 |
| Folic acid (mg/Kg) | 0.5 | 19 |
| Biotin (µg/Kg) | 40 | 310 |
| Choline-Chloride (mg/Kg) | 1600 | 2300 |
| Inositol (mg/Kg) | ND | 80 |
| **Trace elements** | | |
| Iron (mg/Kg) | 280 | 139 |
| Manganese (mg/Kg) | 90 | 82 |
| Zinc (mg/Kg) | 64 | 56 |
| Copper (mg/Kg) | 18 | 12 |
| Iodine (mg/Kg) | ND | 0.97 |
| Selenium (mg/Kg) | ND | 0.13 |
| Cobalt (mg/Kg) | ND | 0.13 |
